# Designing ICTs for Users with Mild Cognitive Impairment: A Usability Study

**DOI:** 10.3390/ijerph17145153

**Published:** 2020-07-17

**Authors:** Diana Castilla, Carlos Suso-Ribera, Irene Zaragoza, Azucena Garcia-Palacios, Cristina Botella

**Affiliations:** 1 Department of Personality, Evaluation and Psychological Treatment, University of Valencia, 46010 Valencia, Spain; 2 CIBER of Physiopathology of Obesity and Nutrition (CIBEROBN), ISCIII CB06/03/0052 Instituto Salud Carlos III, 28029 Madrid, Spain; izaragoz@uji.es; 3 Department of Basic Psychology, Clinical Psychology and Psychobiology, Universitat Jaume I, 12071 Castellón, Spain; susor@uji.es (C.S.-R.); azucena@uji.es (A.G.-P.); botella@uji.es (C.B.)

**Keywords:** usability, speech interfaces, cognitive impairment, ICT, elderly, cognitive decline

## Abstract

Background: Research has supported the cost-effectiveness of cognitive training tools enhanced by information and communication technologies (ICT) in several populations, including individuals with mild cognitive impairment (MCI) and age-related cognitive decline. The implementation of ICTs in this population, however, is sometimes challenging to their cognitive and age characteristics. Ultimately, this might compromise the effectiveness of ICT-enhanced therapies in this population. The aim of this study is to test the usability and acceptability of a Eu_r_opean project prototype for elderly care, in an attempt to explore the ICT design needs of users with MCI. Methods: Participants were 28 individuals aged 58–95 years and with a diagnosis of MCI. Results: The results showed a low perception of peripheral elements and the need to place main interaction elements in the centre of the screen. The correlation between the general level of autonomy (daily life activities) and the ICT autonomy level was significant and positive. The speed of audio help had a significant impact on performance. Conclusion: The present work contributes to the literature on ICT usability needs of users with MCI. Some usability recommendations for designing interfaces for this type of user are provided in the text.

## 1. Introduction

### 1.1. Demographic Changes and Mild Cognitive Impairment

The age profile is expected to change globally in the coming decades. According to the U.S. Census Bureau’s latest ageing report, the population will be much older in 2050. Specifically, in the next 35 years it is expected that the increase in the number of older individuals will be considerably greater than that of the younger population [1]. Consequently, with the ageing of the population, the possibility of an increase in the number of cognitively impaired individuals will also rise.

According to a World Health Organization report dementia and cognitive impairment lead the list of chronic diseases contributing to disability and dependence among older people worldwide. Forty-seven million people suffered from dementia in the world in 2015, and due to the ageing of the population globally, this number is expected to be tripled by 2050. These demographic changes not only have a strong impact on the daily lives of patients and their relatives, but also they have substantial consequences for public finances. Specifically, the estimated global cost of dementia care in 2010 was US$604 billion, and it is projected that its worldwide cost in 2030 could be US$1.2 trillion or more [2].

In the previous paragraph, we have presented the global impact of dementia, which is often characterized by an important cognitive impairment. However, dementia and its associated cognitive impairment do not start abruptly. Conversely, dementia is a dimensional construct that begins with small changes in the brain and symptomatology. In this sense, mild cognitive impairment (MCI) is the clinical syndrome that describes the transitional zone between normal cognitive status and dementia and it is estimated that approximately 10% to 15% of individuals with MCI will develop dementia [3]. Therefore, an important societal goal is to delay the transition between MCI and dementia or at least to reduce the speed of the cognitive decline in patients with MCI. Cognitive training is the most frequently reported form of cognition-focused intervention. It contains sessions involving practice on tasks that target aspects of cognition such as attention, memory, and language [4]. There is now sufficient support for the effectiveness of such training in individuals with MCI [5,6]. However, traditional face-to-face interventions may not always be accessible to the older individuals on a large scale because of accessibility reasons (e.g., having to travel long distances to specialized treatment centres) and limited public economic resources. For this reason, interest in the development of technological applications for the cognitive treatment in older adults is increasing [7]. Several studies have provided evidence about the efficiency of cognitive training tools based on information and communication technologies (ICT) when applied as an adjunct therapy for recovering or improving performance on cognitive skills and self-confidence, as well as for an early intervention in individuals with MCI and age-related cognitive decline [8,9,10,11,12].

In particular, reminiscence therapy appears to be an important therapy in MCI and Alzheimer’s disease. Reminiscence therapy is a non-pharmacological intervention used to prompt past memories with music and old photographs, which also facilitates social interactions and increases self-esteem. The use of ICT seems to be particularly appropriate in this kind of intervention. For example, some studies demonstrate the feasibility of using readily available technology (digital video, images, and music) to produce personalized multimedia biographies that hold special meaning for individuals with Alzheimer’s disease and MCI and their families [13,14,15,16].

### 1.2. Information and Communication Technologies (ICTs) and Cognitive Decline

Rapid technological advances offer an excellent opportunity to face the challenge of promoting independence, strengthening social connectedness, and preventing isolation in older individuals [17,18]. Furthermore, a recent meta-analysis that using computers for leisure produced an overall significant reduction in the risk of dementia [19].

In addition to its recreational role, a systematic review of technology-supported reminiscence therapy also supported the benefits of using technology in the elderly, this time for therapeutic purposes [20]. Some of these benefits include access to rich and engaging multimedia reminiscence materials, opportunities for people with dementia to participate in social interactions and take ownership of conversations, and a reduction in barriers due to motor deficits during interactions with media. Reminiscence therapy interventions based on ICT have also showed their efficacy in depression treatment in the elderly [21]. This is important because several studies have pointed out that depression, social isolation, and loneliness can negatively impact cognitive impairment [22,23,24,25,26]. In a similar line of supporting the benefits of using technology in the elderly, another study suggested that interventions with tailored social networks and social contacts are also needed to increase social contact in the elderly and to help them to delay and cope with cognitive impairment [23].

Cognitive decline in capabilities, such as memory, attention, perceptual speed, or spatial abilities, is part of normal ageing [27], and for this reason, the new age demography brings new challenges related to the way to improve the independence and quality of life of elderly people and, especially, promote their well-being in different ways [28]. New technologies can help to face these challenges, but their utilization in the elderly and specifically in persons with MCI might have some associated challenges. Although technology is increasingly present in everyday life, the elderly usually face usability problems related to the unsuitable design of central features, such as the graphic user interface design and input device choices, to name some examples. On usability tests, elderly users face a greater number of usability problems than young users [29,30,31], and their ICT experience differs not only in terms of their success rate, but also in terms of emotional factors that should be included as an important part of their experience [32,33,34,35]. Often these negative experiences of older users are consolidated into what has been called a technophobia, that is a computer avoidance due to fear or phobia of interacting with computers [36].

Indeed, research has shown that the majority of non-ICT seniors feel “intimidated” and “anxious” about using technology and anticipate that the Internet is difficult to use and to understand [37,38]. In this scenario, a few studies have explored the possible benefits of improving usability by using embodied conversational agents as a form of assistive technology for users with cognitive impairment and the results so far are promising [39]. Nowadays, the technological barrier for elderly users goes far beyond the application design or the individual’s fear of technologies. In this sense, usability for this type of user has to be conceptualized as a more complex problem in which related but different constructs such as web usability and web accessibility should be taken into consideration altogether when designing technological solutions.

Tim Berners-Lee, inventor of the World Wide Web and Director of the World Wide Web Consortium (W3C) states that “*the power of the Web is in its universality. Access by everyone regardless of disability is an essential aspect*” [40]. In this sense, the European Commission defines web accessibility as a policy of e-inclusion that aims *“to allow everyone, including people with disabilities, to perceive, understand, navigate and interact with the Internet*” [41]. In fact, the European Commission went a step further with the development of the Directive (EU) 2016/2102, an important document whose purpose is to ensure digital inclusion and web accessibility by indicating specific standards in the design of websites and mobile apps [42].

Other important contribution that aimed to make webs more accessible to people with disabilities was the Web Content Accessibility Guideline developed by the World Wide Web Consortium (W3C) [43]. The Accessibility Fundamentals summarized by the W3C revealed four relevant issues for older users, namely hearing loss, vision decline, physical decline, and cognitive decline. This important document highlighted that the cognitive decline can affect navigation, comprehension, and task completion due to difficulties with concentration and coping with information overload, distraction from movement or irrelevant material, and short-term memory limitations [44]. In 2019, the W3C developed a more specific section with accessibility standards for users with cognitive or learning disabilities. These include, to name some examples, the need to present content in different ways, make texts easily readable, and provide enough time to read and use content [45].

One key aspect of technology is its connectivity through the Internet. However, the technological characteristics of the Internet (mainly its undefined structure) can be barrier for accessibility and usability of technologies in the elderly. For instance, a meta-analysis [41] revealed negative age effects on spatial abilities, so that time is likely to play an important role in ICT usability. This problem has an important impact when using technologies because the use of the Internet requires spatial abilities due to hypertext characteristics where the user must build the structure of the information or tasks during navigation (e.g., Where was I before? Where should I go now? In which order should I do the required steps?). As a consequence of the previous and the lack of ICT experience, elderly users show better performance on systems with linear navigation [32,46,47]. Another aspect that adds up to the complexity of usability in the elderly lies in the fact that the characteristics of elderly users are not static and vary over time because there are changes due to age-related decline [48].

The aforementioned barriers refer to the elderly in general. Not surprisingly, cognitive impairment in this population makes it even more difficult to use technology. For example, users with cognitive impairment make more mistakes and need more time to use web platforms due to their difficulties in orientation [49]. Thus, it has been suggested that people with mild to moderate cognitive impairment should be offered with simple technologies [50]. In addition to this, visual attention and control of visual short-term memory decline as a result of neurodegenerative processes that occur with ageing, MCI, and AD [51,52,53], which reduces the individual’s ability to respond on a visually dynamic real-world task [54]. Ultimately, this means that, when interacting with computers, users do not behave the way the designers planned [55]. A popular related concept is change blindness, which is defined as the inability to detect changes in visual scenes, in the sense that users focus their attention on an image using visual short-term memory to store relevant information [53]. Because of this decrease in visual attention and control of visual short-term memory, change blindness may be much greater in people with cognitive impairment. Program designers, especially those of young ages, are not likely to be familiar with these characteristics and needs of elderly users. This poses important limitations in the design of technology for this population because taking into account the mental model of individuals with MCI is crucial for the adequate design and testing of user-friendly ICT-based applications and services [56].

Considering the previous, this study aims to analyse the ICT usability needs of users with MCI. Specifically, the purpose of the study is to provide some usability recommendations to design technologies for these users. To carry out this investigation we have used an application named the ehcoBUTLER project, a ground-breaking and comprehensive service solution designed to improve the quality of life of older people by promoting a healthy lifestyle and active ageing through the use of tools that enhance positive emotions and cognitive training [57]. This project has been developed with the support of the European Union’s Horizon 2020 research and innovation programme [58]. The system has been developed following the guidelines of a software that has proven its usability with elderly users using linear navigation [32,46,59]. The study was performed in four iterative cycles to obtain more robust usability recommendations for ICT developments in MCI users.

## 2. Materials and Methods

### 2.1. Design

The study consisted of a classic usability test [60], where the first prototype of the ehcoButler system was used in a controlled environment. The assessment method used was a task analysis [61], applied individually, where the user performed several predefined tasks in order to obtain quantitative data. To carry out this study, only one group was defined. This included users with a diagnosis of cognitive impairment (mild and moderate) made by a physician (neurologist or geriatrician).

The study included performance measures during the test and measurements at pre-test and post-test. The main task consisted of writing an email to a specific recipient and attaching a picture facilitated by the experimenter. As a secondary task, qualitative information was collected about user preferences, iconography, appearance of the avatar, and reality judgments about the synthetic avatar voice.

### 2.2. Participants

The inclusion criteria were: age around 60 years old or more, MCI diagnosed by a geriatrician or neurologist, as well as conserving enough cognitive ability to have a conversation, sufficient hearing capacity, and sufficient visual and motor abilities to interact with the system or with the professional.

The Spanish health care system is universal (all citizens are covered), free of charge, and organized in 17 autonomous communities that apply the General Health Act [62]. The Spanish Ministry of Health, Social Services, and Equality approved the country’s first National Health System strategy for Neurodegenerative Diseases in 2016. It includes early diagnosis strategies and a personalized social and health care plan for each patient [63]. Nursing homes have protocols for cognitive impairment assessment and interventions that are aligned with this national strategy. The users were assessed on cognitive functions (executive functions, episodic memory, visuospatial ability, naming ability, and verbal fluency), functionality (impairment in daily activities), neuropsychiatric symptoms, and biomarkers, by a neurologist or geriatrician, following the guidelines of the “Mild cognitive impairment in the elderly Consensus document” [64].

The final sample consisted of 28 participants with a MCI diagnosis from two nursing homes: 50% men and 50% women, aged between 58 and 95 years, with an average of 76.98 years (*SD* = 9.56).

The literacy level was mostly very basic because 84.6% of the sample only went to school up to the age of 14 (see Table A1). The average age when the sample left school was 11.27 (*SD* = 5.07), with a mean of 5.92 years of school attendance (*SD* = 3.67). Three participants never attended school, even though they could all read and write because they learned at home. Two participants did not recall going to school. This educational level (low or none) is representative of people from 65 to 85 years of age in small cities or towns in rural areas, given that the country had great economic difficulties during the 1940s and 1950s as a result of the Spanish civil war (1936 to 1939). At that moment, most of them left school and went to work at an early age to help their families.

Regarding their previous experience using ICTs, 82.1% had never used a personal computer or a tablet before this study, and only 7.1% of them had used a personal computer or a tablet more than 10 times.

The participants were randomly divided into four groups of seven participants each in order to test the system in four iterations.

Apart from the end-users, three professional caregivers (working at the recruitment centres) took part in the experiment as observers.

### 2.3. Materials

#### 2.3.1. Main Software

The first ehcoBUTLER [58] functional prototype was the main software used for the study. The prototype was developed following all the usability characteristics of the BUTLER system, an emotional and social platform designed for elderly people with low digital literacy skills that has proven its usability and acceptability with elderly people without cognitive impairment [21,46,59,65].

The most important design feature of ehcoBUTLER is the navigation system, which follows a linear structure (i.e., like a step-by-step system) [46]. First, in the main menu, the user selects the application to use. Then, on each step, a human-looking avatar explains where they are and what they can do next. The avatar is “the butler” and its goal is to help them to decide what to do in every step of the system. The avatar is represented graphically as a young man and provides the help through audio (a synthetic voice) and text. The buttons have different colors depending on the type of action they allow: green is for buttons whose actions allow the user to continue with a task; red allows to interrupt a task (i.e., delete data or undo a concluded step); and orange represents secondary actions. Because all the system applications follow the same design principles, the email application was selected for the usability test. A complete description of the email task will be facilitated in the procedure section.

#### 2.3.2. Variables and Measuring Instruments

As we described in more detail below, the assessment protocol consisted of three parts, namely user information (collected before the task), an evaluation of task-related information (performance and user’s opinion), and effectiveness of the task as reported by professional caregivers.

##### User Profile

The user profile data were collected before the usability test. It included demographic data such as sex and educational level. In addition, we evaluated the user’s experience with computers and the Internet, as well as whether the user had an e-mail account and had taken any computer training courses previously.

The level of autonomy in daily life activities was measured based on the professional’s clinical judgement after an interview with the participant. The following item was then responded: “The user can perform his/her daily routines entirely unassisted” with a 5-point Likert scale with response labels: (0) strongly disagree, (1) somewhat disagree, (2) neither disagree nor agree, (3) somewhat agree, and (4) strongly agree.

##### Measures Based on User Performance

Success rate. The success rate was determined in absolute values: 0 (did not successfully perform the task); 1 (successfully performed the task).

Assistance received. The experimenter was not allowed to help the user solve the task. However, participants could be reminded that they could review the avatar’s help, which counted as a new attempt. They could also be reminded about the name of the email recipient or on how to find a letter on the keyboard. Any help was annotated.

Number of attempts. The number of attempts was recorded, based on the number of times the experimenter had to show the user how to review the avatar instructions to find out how to continue.

##### Instruments Measuring the User’s Opinion

Post-test questionnaire for the user: face scales [66] can help to evaluate the mood state in patients with cognitive impairment [67,68]. This questionnaire assesses the user’s opinion of the system, with all the items using a 5-point face scale, rated from left to right with the following labels: strongly disagree, disagree, neither agree nor disagree, agree, and strongly agree. The variables measured were perceived ease of use, learnability and controllability, self-efficacy, flexibility, clear and easy to understand, usefulness, and intention to use.

Finally, a single item using a 5-point face scale to explore the feelings during the test was rated from left to right with the following labels: very bad, bad, neither bad nor good, good, very good.

All the items were obtained from a previous usability study with elderly users (see [32]).

##### Measures Regarding Professional’s Judgment

The NASA Task Load Index–TLX [69] was filled in by the professional caregivers to evaluate the perceived workload. This is done to obtain a measure of the effectiveness of a task. This questionnaire provides an overall workload score based on six subscales: mental demand, physical demand, temporal demand, performance, effort, and frustration. Each scale is divided into 21 degrees from very low to very high. The experimenter trained the professional caregivers before the user test on how to fill in the NASA-TLX properly.

#### 2.3.3. Hardware

**Figure A1:**
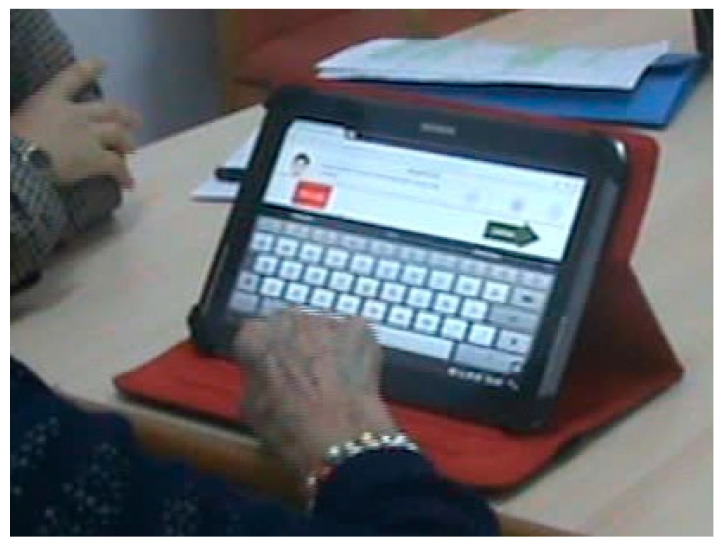
Usability problem on tablets due logic keyboard.

Two set-ups were tested for this study. First, a 10″tablet (*Samsung Galaxy tablet Tab2*) was used because this was originally expected to be a suitable set-up due to its portability and affordable price. However, this was ruled out at the beginning of the study due to additional usability difficulties resulting from the use of the logical keyboard of the device, which hides half the screen during typing processes (see Figure A1). The second set-up which was finally used in the study, was a personal computer with a touch screen and physical keyboard, big keys in ABC order, and a normal keyboard with QWERTY order (*all in one MSI Model AE222-274 G3250 4GB 1TB W10 21.5* inches). A videocamera with a tripod was used to record the sessions.

In addition to the experimenter, the professional caregivers also assessed indicators of performance on the task (whether the user completed the task or not, the number of attempts, the degree of assistance needed during the test, and the technology used). They also reported their clinical judgment about the difficulty of the task, feelings observed in the user, together with suggestions and recommended improvements in the system. Finally, the professional assessed the ability of the user to use the system in an autonomous way (i.e., without any kind of support), using a 5-point Likert scale with response labels 0 = strongly disagree, 1 = somewhat disagree, 2 = neither disagree nor agree, 3 = somewhat agree, and 4 = strongly agree.

#### 2.3.4. Statistical Software

For data analysis, IBM SPSS 22.0 (IBM Corp., Armonk, NY, USA) was used. Descriptive statistics were performed to explore the frequency and percentage of responses for all the variables and a Spearman correlation analysis was conducted to explore the relationship between the user’s level of general autonomy and the number of attempts, as well as the relationship between the user’s level of general autonomy and the extent to which the user could use the system autonomously in the future. A contingency table was created to explore the relationship between the user’s performance and feelings during the task. This was done to explore whether the user’s feelings were related to performance. To investigate whether the non-verbal language displayed by the users matched with their reported feelings, we asked the professionals to rate the observed user’s feelings. Then both ratings were correlated by Spearman analysis. Then, results were graphically represented with Microsoft Excel (Microsoft Corporation, Redmond, WA, USA).

### 2.4. Procedure

To carry out this study, several nursing homes and socio-health organizations dedicated to elderly care in the region of Valencia (Spain) were contacted. We debriefed the managers of the organizations about the objective of the project and the need to test the system with people with MCI diagnosed by a physician (neurologist or geriatrician).

Two collaboration agreements were finally signed between the Jaume I University and two nursing homes from the Health Department of the Valencian Community (Spain). It took two months for the study to be conducted, including the development phases.

The medical staff of both nursing homes selected users with MCI as candidates for the study. Due to the candidates’ cognitive state, the nursing homes contacted their families to obtain their informed consent to participate and permission to video-tape the sessions. For users under the tutelage of the local government (Health Counsel of the Valencian Community), an additional permit was required to carry out the study. The research was conducted following the American Psychological Association’s ethical principles and code of conduct [70]. The study was approved by the Ethics Committee of the Gestión Sociosanitaria del Mediterráneo (GESMED, Socio-Sanitary Management of the Mediterranean; REC number: CD643566/2017). The participants’ data confidentiality and anonymity were ensured. Forty-six users were asked to participate in this study voluntarily. Of these, 33 agreed to participate and 28 met the inclusion criteria and signed the data and recording confidentiality agreement. The experimental session consisted of a usability test [60], where a task analysis was carried out [61]. The experimenter took all the necessary hardware to be used in the study to the nursing homes. The rooms used in the centres were big enough to place the equipment and facilitate the movement of people with reduced mobility.

The centre’s psychologists (the professional caregiver) accompanied the participants while the expert in usability (the experimenter) conducted the tests. Next, the experimenter presented the task and administered the pre-test assessment protocol (demographic data and previous experience with computers and the Internet). Once the questionnaires were completed, the experimenter explained the task to the user, gave them the instructions for the task on paper, and asked if they would prefer to use a physical or a logical keyboard.

The main task involved writing an email to a specific contact already registered in the address book and attaching a specific picture using the ehcoBUTLER system. The user had the instructions on paper, with the name of the contact in red, the text of the email, and the image to be attached, so that they could follow the instructions as easily as possible and could effectively perform the task. Therefore, the user did not have to memorize the instructions. The secondary task required the evaluation of the avatar and the help voice from a qualitative point of view. We also asked the participants to explain the reasons for their preferences. In order to obtain reliable data on task performance, the sessions were video-taped. A usability expert reviewed the sessions.

**Figure A2:**
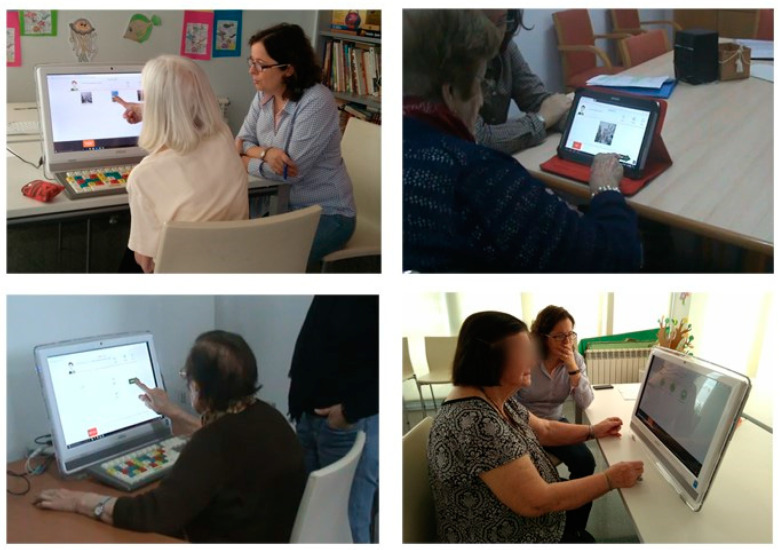
Set-up during usability testing.

The role of the experimenter was to encourage the users to perform the task autonomously, without providing additional instructions. If the users required assistance, the experimenter encouraged them to interact with the avatar. Success meant that the user could complete the task without the help of the experimenter. If any kind of help was needed to complete the task, this was noted as failure. The number of attempts was recorded according to the number of times the experimenter had to show the user how to review the avatar instructions to find out how to continue. The experimenter stood next to the users throughout the usability test (see Figure A2).

**Figure A3:**
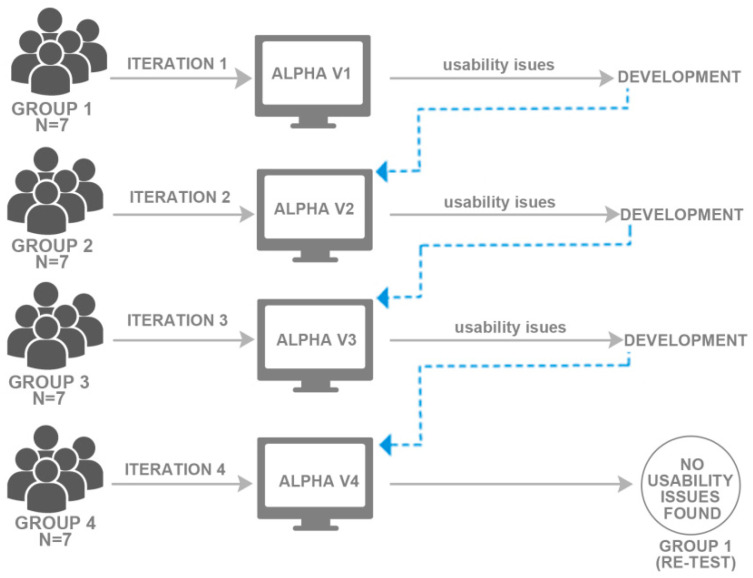
Diagram of iterative cycles performed in the usability test.

In accordance with the Iterative User Interface Design [60], the study was divided into four iterative cycles. This began with the first functional prototype of the system and included seven new users in every iteration. Usability issues were found in each iteration and the proposals for improvement were implemented by the development team. In each iteration the usability issues found were fixed, and after tested again, until arriving to iteration 4, where no usability problems were found. Finally, a re-test was carried out with the users from iteration 1 (see Figure A3). Finally, after finalizing the test, the post-test questionnaires were answered by the users with the experimenter’s support (their opinion about the system and how they felt during the test). The professional’s clinical judgment was also recorded at this stage. As noted earlier, this included whether the user completed the task, the number of attempts, the assistance received, the opinion about the system and the technology used, the workload experienced, and suggestions and recommended changes.

## 3. Results

### 3.1. Main Usability Findings

#### 3.1.1. Solved through Behavior Program Changes

**Figure 1:**
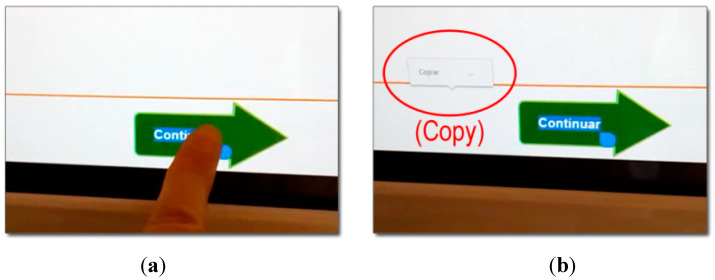
Troubles found due to very long pulsations. (**a**) Text label button selected due to very long pulsation. (**b**) Secondary button activated (copy) instead clicking on the main action button (the green arrow).

An important problem was related to the way in which users pressed on the screen. The users performed very long pulsations (an average of 3–4 s pressing), which led to the following problems: instead of clicking on the main button (e.g., continue), the secondary button (e.g., copy) was activated. Thus, the text label button was copied, as shown in Figure 1, as opposed to following to the next action.

This problem was solved by writing a piece of code that overrides the behaviour in the web browsers related to the right-click mouse events by converting these events to left-click mouse events.

Another usability issue observed was the user’s need to see what they are pressing while interacting with the touch screen. As a result of this, they clicked outside buttons. To solve this problem, we programmed an interaction area higher than the visual button only, thus preventing missclicking for this reason.

Regarding the help audio facilitated by the avatar voice (a synthetic voice), we found problems with standard speech speed (around 3 words per second). Some participants had problems following the instructions because these were too fast for them. The speed was changed to 2 words per second, adding 8 spaces (2 s) between sentences in order to clearly separate the concepts in the instructions. For example: “You are now viewing your letter (2 s of silence). If you like it (2 s of silence), press the green button that says: Send letter”. After changing the voice speed, 92% of the users informed that they liked the voice and that it was easy to understand.

#### 3.1.2. Solved through Graphical Changes

**Figure 2:**
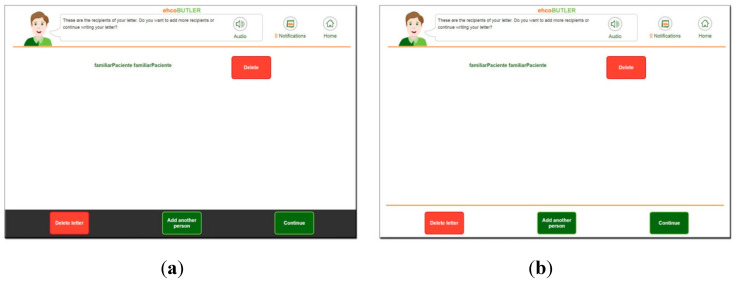
Graphic user interface (GUI) colour change in bottom interaction area. (**a**) Original design. (**b**) Modified design.

The main usability finding we encountered related to the cognitive state and mental model of this type of user had to do with their attentional capacity. A large number of users did not see the interaction buttons during the first usability iteration. Our first hypothesis was that maybe they suffer some cognitive blindness to bottom elements, which might be caused by the dark grey colour of the graphic user interface (GUI). This is shown in Figure 2a. Because we anticipated that some users might perceive that the dark grey band was an external element, the graphic user interface was redesigned and the dark grey colour was replaced by an orange line, following the upper design as in Figure 2b.

**Figure 3:**
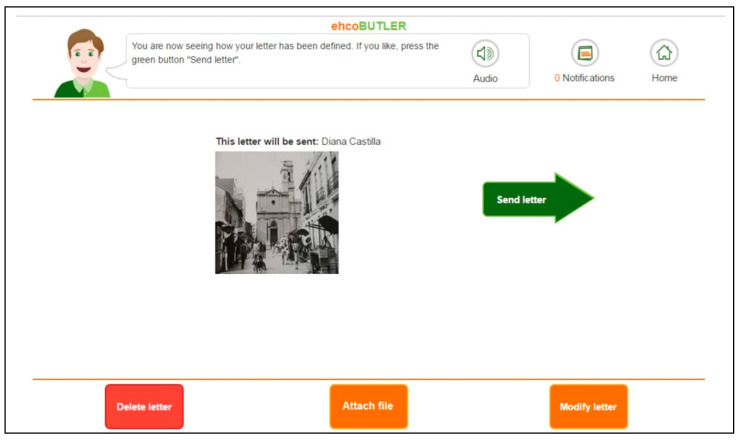
Final graphic user interface.

Despite this change, users still did not see the elements at the bottom. The study of the sessions revealed that the less autonomous users presented a very acute attentional focus towards the centre of the screen, to the detriment of the interaction elements placed at the bottom or sides of the screen. In order to solve this problem, the graphic user interface was redesigned again, placing the main interaction elements in the central area, that is, within the attentional focus of these users (see Figure 3). With this change, the interaction issues were fully resolved.

### 3.2. Quantitative Results

#### 3.2.1. Task Performance

Good results were obtained on task performance. Specifically, 89% of the participants successfully completed the task (even though the majority of them had no previous ICT experience).

#### 3.2.2. Number of Attempts

The number of attempts to complete the task varied across participants (range 1 to 6). However, 50% of the users managed to complete it in just 1 or 2 attempts (median = 2.50, *SD* = 1.59). Comparing the sum of attempts during the study, we observed that there was a higher number of attempts at the beginning of the study due to usability problems related to the touch interaction (long pulsations that activate selecting text or the contextual menu). We conducted a Spearman correlation analysis between the user’s general level of autonomy, rated by the professional caregivers, and the number of attempts. The correlation was not significant (*r_s_* = 0.17, *p* = 0.396).

#### 3.2.3. Workload NASA Task Work Load Index)

**Figure 4:**
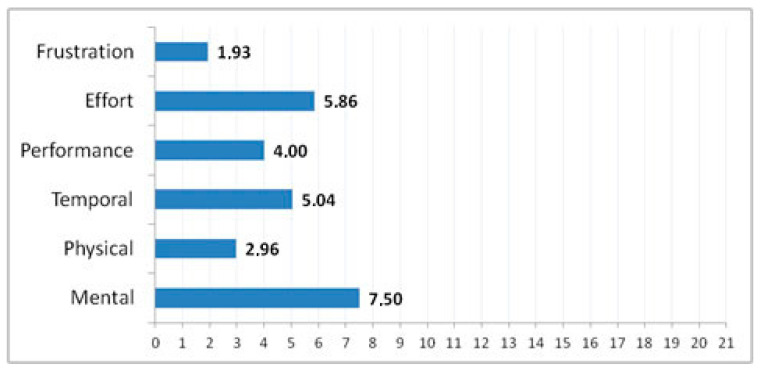
NASA task work load index results.

The workload during the task was low or very low on all subscales (see Figure 4), achieving a general mean of 4.55 (*SD* = 5.65) and means on all scales below the central point of 11 (range 0 to 21, Very low demand = 0; Very high demand = 21).

### 3.3. Quantitative Results

#### 3.3.1. Usability Variables and User Opinion

**Figure 5:**
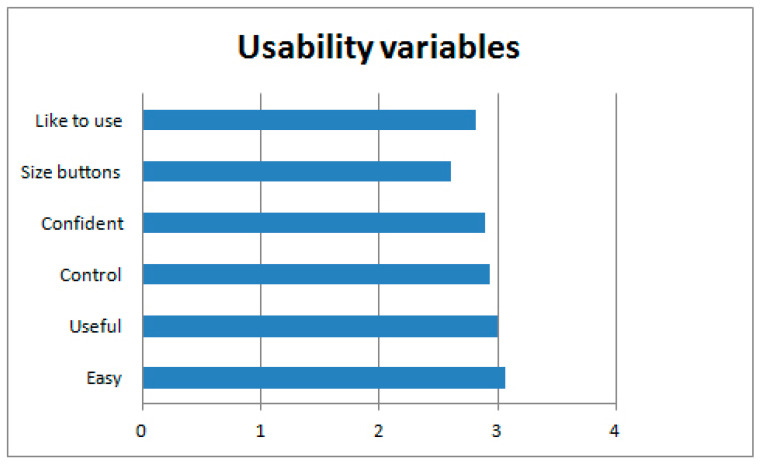
Average of usability variables.

In general, the users’ opinion was good, as all the usability variables were scored on the positive side of the scale exceeding the midpoint (i.e., exceeding 2 points on a scale ranging from 0 to 4). Specifically, the users found the system easy to use and useful for their lives, they felt confident while using it, felt that they had control over the system, found the button size large enough to see it and interact with it, and reported that they would like to use the system in the future (see Figure 5).

The variable with the highest average score was ease of use, with 3.07 (*SD* = 0.98), followed by useful with 3.00 (*SD* = 1.19).

Regarding their feelings during the test, 79% of the users felt from normal to very good. A contingency table was created to explore the relationship between user performance and feelings during the task (see Table 1).

Only three users (10%) referred to feeling bad during the experiment. They indicated that they felt nervous because of the test conditions (people looking at them and assessing what they were doing). The number of users who felt bad or failed the task were too low to perform a Chi-square analysis.

#### 3.3.2. Intention to Use

Regarding the intention to use the system in the future, 82% of the users expressed that they would like to use it often in the future (21% strongly agree; 61% somewhat agree; 7% neither disagree nor agree; 0% somewhat disagree, 11% strongly disagree).

#### 3.3.3. Preferences about Avatar Appearance and Voice

From a graphic point of view, users indicated that they liked the age of the avatar (young), estimating that it was about 30 years old. Regarding the voice, none of the participants perceived that it was a synthetic voice. Adjectives such as “nice”, “polite”, or “kind” were used the most to describe it.

### 3.4. Professional Opinion

#### 3.4.1. Ability to Use the System in an Autonomous Way

Before the experiment, the professional caregivers rated the level of autonomy the users had in their everyday lives while performing any kind of task.

After the usability task, the professional caregivers also rated their clinical judgment about the level of user autonomy using the system, revealing that 46% of the users would be able to autonomously use the system in the future without any kind of support. Only in 14% of the cases did the professional caregivers describe that the users would need frequent support, but they felt that 39% of the sample would always need support.

We conducted a Spearman correlation analysis between the user’s general level of autonomy, rated by the professional caregivers and the extent to which they thought the user could use the system autonomously. The correlation was significant and positive (*r_s_* = 0.45; *p* = 0.016). This correlation indicates that the professional caregivers thought that users with a high degree of autonomy in their everyday lives could also use the system in a more autonomous way in the future, whereas users with low autonomy in their routines would also have low autonomy in using the system. It seems logical that if the user is not autonomous in his/her daily life, it would be very difficult for him/her to use the system without assistance.

#### 3.4.2. Sessions Needed to Learn to Use the System in an Unassisted Manner

**Figure 6:**
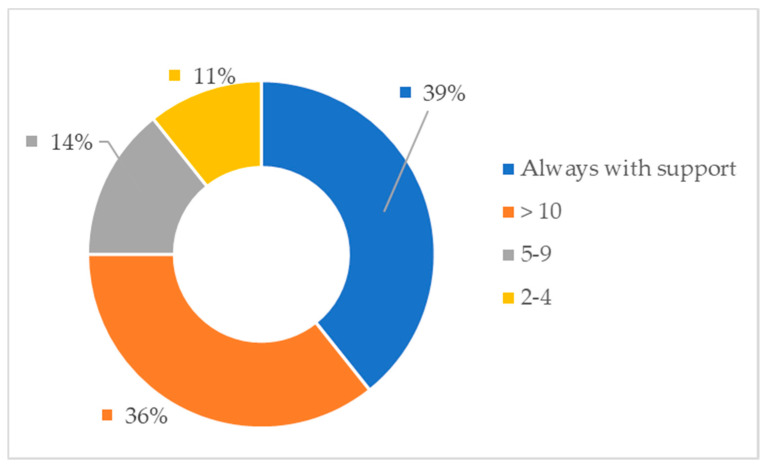
Estimated number of training sessions to use the system in an autonomous way.

The following analysis evaluates to which extent the professionals believed that the users would be able to use the system on their own with some training. According to the professionals’ opinion, 39% of users would always need support to use the system. The also indicated that 36% of users would need more than 10 training sessions to use the system on their own. Finally, they reported that 14% of them would need between 5 and 10 training sessions to use the system autonomously and 11% of them would only need 2 to 4 training sessions (see Figure 6).

#### 3.4.3. Professional Opinion about the Users’ Feelings

The professionals also rated how they thought the users felt during the test. We conducted a Spearman correlation analysis between feeling scores given by the user and those given by the professional. The correlation was significant and positive (*r_s_* = 0.59; *p* = 0.001), indicating a large agreement between the experienced user feeling and the professional caregiver’s impression about the user’s feeling.

## 4. Discussion

The present study aimed to test the usability of a prototype system designed for users with MCI and make adaptations to it based on the results. For this purpose, a functional prototype email system was designed based on the usability design principles of the Butler system (i.e., the design followed the National Institute on Aging checklist [71] and added linear navigation and audio and text help through an avatar) [59]. The system was tested in two nursing homes of the Health Council of the Valencian Community (Spain). The results showed differences between the present study and the findings obtained from previous research with the same web design but a different population (i.e., people without cognitive impairment) [32,46]. The main usability issues revealed in the present study compared with those of users without MCI were the following:

- *Related to spatial abilities and attention*. The results showed a low perception of peripheral elements and the need to place the main interaction elements (e.g., continue the action in the step-by-step navigation) in the center of the screen because less autonomous users showed an attentional focus on this central part of the screen and attentional blindness to the peripheral graphical elements.
- *Related programming interaction*. Given that users expect a real time change in buttons (such as sinking down when pressed), the interaction with the buttons was performed by pressing them for a long time, causing an unexpected interaction result (copying text or activating the secondary browser menu). On the other hand, we observed the user’s need to see what they were pressing while touching the buttons. This explains why they clicked outside the buttons.
- *Related audio help.* The standard speed of synthetic speech (3 words per second) was too fast, and most of the users were not able to follow the instructions.

Our findings raise some novel questions about the design of interfaces for MCI users. For example, several studies point out that the use of audio and text support can benefit both novel and experienced older users [72]. However, audio interfaces also could represent a new usability barrier because they force the user to work with memory and mental agility. These new interfaces allow us to avoid the keyboard and present a user interface with less text, but the audio interface adds other difficulties such as having to understand and retain information while making interaction decisions. It is well known that, because speech is linearized, audio instructions as a unimodal strategy impose difficulties in the elderly [73]. In the case of cognitively impaired users, this effort could become another step in the technological barrier stairway. If we take into account that one of the handicaps in MCI is memory loss and a decrease in mental agility, we could encounter a real barrier that goes beyond usability and could become a serious accessibility problem for this kind of user.

Regarding the audio interface to support the interaction, previous studies with elderly users without cognitive impairment suggest that speed adjustment is not necessary [72]. However, our findings showed that it is likely to be necessary to adjust the speed of the speech, as well to add a short time separation between phrases for users with MCI, probably because they need more time to process information. Our study revealed positive results from adjusting the speech speed to 2 words per second and adding 2 s of silence between short sentences, which was done to clearly separate the concepts of the instructions and to give the user time to understand them. At this point, we think it is important to differentiate between input and output audios. Our study only explores this feature as an output of an application, that is, when the user receives information passively (in our case as a complement of the text help). We cannot extrapolate our conclusions to audio inputs, that is, when the user gives audio instructions to the system.

In addition to the findings with the audio interface, our results showed usability difficulties related to the graphic design. A metaphor in this context is how we represent an interaction with the system in an understandable way. For example, a metaphor in a graphic interface could be represented by the buttons and other visual elements like the avatar or the space to write. The commonly used metaphors represented in the technology do not correspond to the previous experience of users with a low technological profile and according literature this can be a barrier [59,74]. This might be the reason why the users in our study made long pulsations while waiting for a change of state in the buttons. These results support the idea that assistive technologies should introduce graphic elements that match the users’ previous experience. This opens an avenue for a new dimension that should be considered in the design of this type of technology. Following this need to adapt ICTs to the mental model of the elderly, it could be advisable to represent objects and object behaviours that users could be familiar with. For example, if we represent a button in an application, the feedback when pressing it could be seeing the button sink.

Previous literature highlights the negative effects of age on spatial abilities, memory, attention, and perceptual speed [19,27,75]. Thus, because navigation is one of the greatest difficulties for older users, linear navigation can be useful for improving usability results with elderly users [46,76,77]. Experiencing MCI considerably increases these cognitive and motor difficulties, which adds new challenges in designing webs for this kind of user. Some studies propose linear navigation to avoid spatial disorientation when using websites [75]. However, cognitive impairment influences spatial orientation and, consequently, makes this usability problem even greater. The linear navigation solution cannot fix this because, for users with MCI, spatial orientation might not be limited to navigation among the different parts of the system. The problem could be that, on each screen, the user must make an effort that requires attention and guidance. This idea seems somewhat aligned with the change blindness concept [55] because the users could not find the changes between different screens, and so there was disorientation beyond navigation. A possible solution is to combine different strategies to maintain the attention on interaction effortlessly. For example, one option could be combining the linear navigation with audio and help text and a design in which the main interactions (to continue the task) are placed in the centre of the screen.

Also in relation to cognitive performance, research has evidenced that, when the user is familiar with the learning object, the memory and learning processes are faster [78]. However, cognitive impairments such as memory loss or disorientation could increase the difficulties in recognizing or memorizing objects. Natural interfaces such as speech mode try to replicate a natural and user-friendly interaction. Audio outputs (from the system to the user) can be useful for users who receive help and instructions in real time [72], but audio inputs (from the user to the system) require other mental processes that demand the specific use of memory, such as remembering specific words from the instructions or predicting (or remembering) what step will be next. In this regard, a recent study [39] evidenced significant difficulties in speech recognition in patients with MCI even when embodied conversational agents were successfully implemented. Our study only explored audio outputs, and the results showed that the speech speed could be an important factor in designing audio interfaces.

Another finding was that a positive and significant correlation between the general level of autonomy and the ICT autonomy level was obtained. This result could indicate that the level of autonomy in everyday activities could be a good predictor of the level of user autonomy in the use of assistive technologies, which might have some clinical implications. For example, this could be used as guiding information when having to select the type of interface or amount of support that is provided to users (e.g., those with less daily autonomy should be offered more help or easier interfaces).

From a qualitative point of view, the results of our study showed that users did not perceive the difference between a synthetic voice and a human voice during the testing. These results are important as they suggest that this system can be effectively used in the elderly, which is in line with past literature [72]. This finding supports the idea that the use of mechanical audio interfaces, which are cheaper to develop than audios recorded directly by humans, are suitable for this kind of population. Given that this was perceived like a human voice and did not eliminate emotional qualities, this procedure is promising in this field.

The present study has a number of limitations. First, although the sample size is large enough to obtain usability conclusions, the number of users limits the statistical analysis that can be performed with the data. Additionally, the fact that they all belonged to the same country limits the cultural generalization of the findings. Furthermore, the experimental design limits our conclusions to the specific task of “composing” an email and attaching a picture. Other tasks and interfaces should be used to test our usability recommendations for MCI users. An additional limitation refers to the existence of a single professional rater of the patient’s performance. Even though this is the usual practice in clinical settings, the existence of a second evaluator is always preferable for reliability purposes. Finally, this work did not explore in-depth the impact of physical or logical keyboards on performance.

## 5. Conclusions

In spite of the limitations of this study, the findings resulted in a number of recommendations for the use of ICT in persons with MCI and open the door to exploring new dimensions of spatial orientation on graphic user interfaces. New technologies can help to improve the wellbeing and quality of life of users with MCI or early dementia. However, due to their cognitive impairment, such individuals suffer from a lack of orientation, not only when navigating between screens, but when interacting with a single screen. Simplifying technologies for MCI users should be an important societal goal. The use of linear navigation could be a key element in this direction, but it is not the only one. Designers have a new challenge of “designing interaction in the middle” because the main area of attention for these users was the centre of the screen. Another recommendation is to design the interaction area bigger than the visual button. This should be done because there is a general tendency to press the buttons outside the image in an attempt to see what they are pressing. In addition, the results suggested that natural interfaces, such as audio interfaces, might be useful for these users if the speed is adapted to their cognitive needs. In this sense, text-to-speech technology can be a suitable and cost-effective alternative because users do not distinguish the human voice from a bot. Therefore, based on our findings one recommendable speech speed for similar users and purposes would be 2 words per second and adding 2 s of silence between short sentences. Ultimately, the utility of the results lies in the fact that achieving design interfaces that give MCI users independence could help to prolong the time they live independently at home and might help improve their quality of life. One way of doing this, for instance, would be providing them with a number of alternative care possibilities, such as a cognitive rehabilitation program that they can perform at home autonomously. The main contribution of this study consists of exploring the usability needs of users with MCI on ICT systems and providing some usability recommendations for designing interfaces for this kind of user.

## Figures and Tables

**Table 1:** User performance vs. feelings during the task: contingency table.

		Task Performance		Total
		Fail	Success	
**User Felt**	Very bad	0	0	0
	Bad	2	1	3
	Neutral	1	4	5
	Good	0	15	15
	Very good	0	5	5
**Total**		3	25	28

## Appendix A

**Table A1:** Summary of sociodemographic characteristics of the sample.

ID	Number of Iteration	Cognitive Diagnosis	Age	Sex	School-Leaving Age	PC Experience
1	1	MCI	73	M	12	Never
2	1	MCI	83	F	10	Never
3	1	MCI	85	M	Didn’t remember	Never
4	1	MCI	71	F	14	Never
5	1	MCI	94	M	14	Never
6	1	MCI	88	M	10	Never
7	1	MCI	95	F	10	Never
8	2	MCI	67	M	14	Never
9	2	MCI	58	M	13	Once
10	2	MCI	89	F	8	Never
11	2	MCI	90	F	14	Never
12	2	MCI	86	F	Didn’t go to school	Never
13	2	MCI	89	M	Didn’t go to school	Never
14	2	MCI	85	F	Didn’t go to school	Never
15	3	MCI	76	F	8	Never
16	3	MCI	70	M	14	Never
17	3	MCI	85	M	7	Never
18	3	MCI	71	F	13	Never
19	3	MCI	69	M	15	Never
20	3	MCI	75	M	Didn’t remember	More than 10
21	3	MCI	76	M	14	From 1 to 10
22	4	MCI	81	M	12	Never
23	4	MCI	70	F	16	More than 10
24	4	MCI	88	F	12	Never
25	4	MCI	67	M	20	From 1 to 10
26	4	MCI	81	F	18	Never
27	4	MCI	91	F	11	Never
28	4	MCI	78	F	14	Never

Notes: MCI: Mild Cognitive Impairment. PC experience: number of times that the person used a personal computer before this study. Sex: M = Male, F = Female.
